# Bioavailability of Iron, Zinc, Phytate and Phytase Activity during Soaking and Germination of White Sorghum Varieties

**DOI:** 10.1371/journal.pone.0025512

**Published:** 2011-10-07

**Authors:** Abd El-Moneim M. R. Afify, Hossam S. El-Beltagi, Samiha M. Abd El-Salam, Azza A. Omran

**Affiliations:** 1 Department of Biochemistry, Faculty of Agriculture, Cairo University, Cairo, Egypt; 2 Department of Crops Technology, Food Technology Research Institute, Agricultural Research Center, Cairo, Egypt; University College London, United Kingdom

## Abstract

The changes in phytate, phytase activity and *in vitro* bioavailability of iron and zinc during soaking and germination of three white sorghum varieties (*Sorghum bicolor* L. Moench), named Dorado, Shandweel-6, and Giza-15 were investigated. Sorghum varieties were soaked for 20 h and germinated for 72 h after soaking for 20 h to reduce phytate content and increase iron and zinc *in vitro* bioavailability. The results revealed that iron and zinc content was significantly reduced from 28.16 to 32.16% and 13.78 to 26.69% for soaking treatment and 38.43 to 39.18% and 21.80 to 31.27% for germination treatments, respectively. Phytate content was significantly reduced from 23.59 to 32.40% for soaking treatment and 24.92 to 35.27% for germination treatments, respectively. Phytase enzymes will be activated during drying in equal form in all varieties. The results proved that the main distinct point is the change of phytase activity as well as specific activity during different treatment which showed no significant differences between the varieties used. The *in vitro* bioavailability of iron and zinc were significantly improved as a result of soaking and germination treatments.

## Introduction

Sorghum (*Sorghum bicolor* L. Moench) is a crop that is widely grown all over the world for food and feed. It is one of the main staples for the world's poorest and most insecure people in many parts of the developing world, especially in the drier and more marginal areas of the semi-tropics [1]. In these areas sorghum serves as the principal form of protein and energy for several hundred million people [2].

The nutrient composition of sorghum indicates that it is a good source of energy, protein, carbohydrate, vitamins and minerals including the trace elements. Sorghum grain contains 1.3 to 3.3% of ash and minerals such as phosphorus, potassium and magnesium in varying quantifies. Sorghum is also an important source of some minerals, particularly iron and zinc, but all except finger millet is low in calcium [3].

Iron (Fe) and zinc (Zn) are essential trace elements in human nutrition and their deficiencies are major public health threats worldwide. Among the micronutrient malnutrition situations afflicting the human population, Fe and Zn deficiencies are of major concern not only because of the serious health consequences they may have, but also because of the number of people affected worldwide particularly in Africa [4].

Sorghum nutritional quality is dictated mainly by its chemical composition and the presence of anti-nutritional factors, such as phytate. Phytate or Phytic acid is a principal storage form of phosphate, ubiquitously distributed in plants, particularly in cereal grains and in legumes. The effects of phytate in human and animal nutrition are related to the interaction of phytic acid with proteins, vitamins and several minerals, and thereby restrict their bioavailability [5]. In view of the anti-nutritional effects of phytate, many attempts many attempts were carried to reduce it. Other attempts to reduce the phytate content such as fertilisation [6]. These include activation of the indigenous enzyme phytase and/or addition of microbial phytase [7]. Because phytate is water soluble, a significant phytate reduction can be realised by discarding the soak water. Soaking usually forms an integral part of processing methods such as germination, fermentation, cooking and the toasting. Soaking media include water, salt or combination of salts and alkali [8]. Temperature and pH value have been shown to have a significant effect on enzymatic phytate hydrolysis during soaking. If the soaking step is carried out at temperatures between 45 and 65°C and pH values between pH = 5.0 and 6.0, which are close to the optimal conditions for phytate dephosphorylation by the intrinsic plant phytases, a significant percentage of phytate (26–100%) was enzymatically hydrolysed [9].

Germination is a process widely used in legumes and cereals to increase their palatability and nutritional value, particularly through the breakdown of certain anti-nutrients, such as phytate and protease inhibitors. In non-germinated legume grains and cereal seeds, with the exception of rye and to some extent wheat, triticale and barley, only little intrinsic phytate-degrading activity is found [10], [11], but during germination a marked increase in phytate-degrading activity with a concomitant decline in phytate content was observed [12], [13]. Long periods of germination periods are needed to improve mineral bioavailability through germination. The objective of this study was to eliminate the phytate content associated with sorghum grain and improve iron and zinc bioavailability by using simple methods.

## Materials and Methods

### Materials

#### Samples and chemicals

Pepsin, pancreatin, lipase and Cetylpyridinium bromide were purchased from Sigma– Aldrich Chemical Co. (St. Louis, USA) and bile extracts from Win Lab Laboratory chemicals reagents (Mumbai, India). All other chemicals used were of analytical reagent grade.

Three white sorghum varieties (*Sorghum bicolor* L. Moench), grown during the 2007 season were obtained from the Crops Research Institute, Agricultural Research Center for Shandweel-6, and from Central Administration for Seed Certification (CASC), Ministry of Agriculture and Land Reclamation, Giza, Egypt for Dorado and Giza-15. The grains were carefully cleaned and freed from broken seeds and extraneous matter.

#### Soaking of grains

Sorghum seeds were soaked in distilled water for 20 hours with a ratio 1∶5 w/v and the soaked water changed twice. At the end of soaking period, the soaked water was discarded. The seeds were rinsed twice in distilled water and the grains were dried at 45±5°C. The grains were milled in a Laboratory mill to obtain fine flour and kept at −20°C until analysis.

#### Germination of grains

Soaked seeds were germinated for 72 hours at room temperature, and then the grains were dried. The root portions were manually removed. The grains were milled into fine flour and kept at −20°C until analysis.

### Chemical analysis

#### Iron and zinc determination

Total Iron and zinc content were determined according to the method outlined in A.O.A.C [14] by using the Perkin Elmer (Model 3300, USA) Atomic Absorption Spectrophotometer. Approximately 2 g sample was weighed and heated at 550°C. Then the ashes were dissolved with hydrochloric acid 1 M.

#### Phosphorus and Phytate determination

Total phosphorus (TP) was determined by the method of Trough and Mayer [15]. Phytate was extracted according to the procedure described by Mohammed *et al*. [16]. 1.0 g Sample was extracted with 3% tri-chloro acetic acid (TCA) at 37°C for 45 min. with simple shaking followed by centrifugation and extractation by using anion exchange column. The extracted phytate (0.2 ml) was mixed with 4.6 ml of distilled water and 0.2 ml of chromogenic solution and the tubes were heated in a water bath at 95°C for 30 min, and then were allowed to cool. The developed color was read at 830 nm against blank. Standard phytate solution was prepared by dissolving sodium phytate in distilled water to prepare different phytate concentrations as described above in the tested samples. The amount of phytate in the tested samples was expressed as mg phytate/100 g sample.

### Phytase activity assay

#### Extraction of phytase

Phytase activity assayed according to the procedure described by Barrientos *et al*. [17] and modified by Jog *et al*. [18]. Sample (2 g) was added to ice cold Buffer (16 ml of 10 mM Tris–HCl, pH 7.0, containing reduced glutathione, 0.5 mM). The suspension was stirred with a glass rod. Solid cetylpyridinium bromide (80 mg, finalconcentration 0.5% w/v) was added to the suspension. The suspension was homogenized with homogenizer at 27,000 rpm for 2×1 min. with a 1 min delay in-between. The resulting crude homogenate was centrifuged at 10,000 *g* for 30 min. The supernatant containing phytase activity was collected.

#### Alkaline phytase assay

Alkaline phytase activity was assayed by measuring the inorganic phosphate (Pi) released by the enzyme. The assay mixture contained Tris–HCl buffer (100 mM, pH 8.0), NaCl (0.5 M), CaCl_2_ (1 mM), sodium phytate (1 mM), NaF (10 mM), and an aliquot of enzyme solution in a total volume of 250 µl. The assay mixture was incubated at 37°C for 1 h and the reaction was stopped by the addition of 50 µl of 50% TCA. In brief, ammonium molybdate solution (700 µl of a 1∶6 solution of 10% w/v ascorbic acid and 0.42% ammonium molybdate (w/v) in 0.5 M H_2_SO_4_) was added and the solution was incubated at 37°C for 1 h. Absorbance at 820 nm was measured and the inorganic phosphate concentration was determined from a calibration curve using KH_2_PO_4_ as the standard. One unit of enzyme is defined as the amount of enzyme that releases 1 µmol of Pi from sodium phytate per minute under these conditions.

#### Acid phytase assay

Acid phytase activity was assayed in a solution containing sodium acetate buffer (100 mM, pH 5.0), sodium phytate (1 mM), and CaCl_2_ (1 mM). NaF was not added to this assay mixture. The assay mixture was incubated at 37°C for 1 h and the reaction was stopped by the addition of 50 µl of 50% TCA. Pi released in the reaction was quantified as described above. Soluble protein was determined according to Lowry *et al* [19] and specific activity was defined as unit per milligram protein.

#### *In vitro* availability of iron and zinc

The bioavailability of iron and zinc was determined by the *in vitro* digestion method described by Kiers *et al*. [20]. Triplicate samples of sorghum whole meal (5 g) were suspended in 30 ml distilled water and digested under simulated gastro-intestinal conditions, subsequently using α-amylase solution, stomach medium consisting of lipase and pepsin, and pancreatic solution consisting of pancreatin and bile. After digestion, the suspension was centrifuged at 3600 g for 15 min. The supernatant was decanted and the pellet was discarded. The supernatants were pooled and filtered through a 0.45 mm pore filter. A blank was included consisting of 30 ml distilled water digested and filtered as described above. Both filtered supernatants from sample and blank were analyzed for Fe and Zn. Samples were corrected for added reagents/water by subtracting Fe and Zn content of blank from that of supernatants from samples. Iron and zinc content were measured by using the Perkin Elmer (Model 3300, USA) Atomic Absorption Spectrophotometer. The amounts of Fe and Zn (in supernatant were regarded as soluble minerals. Percentage of soluble mineral was calculated as bioavailability %.

Bioavailability % = amount of Fe or Zn (supernatant) – amount of Fe or Zn (blank)\amount of Fe or Zn (undigested sample) ×100.

### Statistic analysis

For the analytical data, mean values and standard deviation are reported. The data were analyzed using the one-way ANOVA model was used applying the LSD test to evaluate significant difference among means at *P*<0.05.

## Results and Discussion

### Changes in iron, zinc and phytate content, phosphorus during soaking and germination of whole grains

From Table 1, it could be noticed that the Fe content ranged between 5.54–7.65 mg/100 g raw sorghum, while the Zn content ranged between 3.99–5.02 mg/100 g raw sorghum, these finding are in agreement with the findings of Jambunathan [21] who reported that Fe content ranged between 2.6–9.6 mg/100 g in samples of about 100 varieties of sorghum. The same result was observed by Kayodé [4] who reported that Fe concentration of the sorghum grains ranged from 3.0 to 11.3 mg/100 g. The Zn concentration ranged from 1.1 to 4.4 mg/100 g. In general, cereals high in phytate tend to have higher iron content. Low extraction (white) flour contains less phytate and iron, while high extraction (brown) flour has both more phytate and more iron.

**Table 1:** Changes in iron and zinc during soaking and germination of whole grains (mg/100 g DW)*.

Treatments	Fe	% Fe loss	Zn	% Zn loss
**Raw**				
Dorado	7.65±0.71^a^	-	4.43±0.05^ab^	-
Shandaweel-6	6.84±0.32^ab^	-	5.02±0.25^a^	-
Giza-15	5.54±1.82^bc^	-	3.99±0.49^bc^	-
**Soaking**				
Dorado	5.19±0.08^cd^	32.16	3.78±0.33^bcd^	14.67
Shandaweel-6	4.10±0.17^cde^	40.06	3.68±0.48^bcd^	26.69
Giza -15	3.98±0.60^cde^	28.16	3.44±0.02^cd^	13.78
**Germination**				
Dorado	4.71±0.40^cde^	38.43	3.34±0.03^cd^	24.60
Shandaweel-6	4.16±0.87^cde^	39.18	3.45±0.32^cd^	31.27
Giza-15	3.41±0.39^e^	38.45	3.12±0.59^d^	21.80
**L.S.D**	1.3281		0.7412	

*Values are mean of three replicates ±SD, number in the same column followed by the same letter are not significantly different at *p*<0.05.

After soaking, the Fe content of the sorghum was significantly lower than raw sorghum (P<0.05). After soaking, the losses of Fe contents were between 28.16 and 40.06%. These finding are in contrast with the findings of Lestienne *et al*. [22] who reported that up to 40% of Fe content of sorghum grain may be lost as a result of soaking. As for germination, the Fe content of the sorghum was significantly reduced by 38.43 to 39.18% (P<0.05).

Lestienne *et al*. [22] found that the zinc content also decreased significantly, but the reduction did not exceed 30% except on Zn content of Shandweel-6. Reduction after soaking may be attributed to leaching of iron and zinc ions into the soaking medium [23]. The leaching of zinc was lower than iron and this phenomenon may be due to the fact that zinc and iron are not located in the same place in the seeds nor are they linked with the same molecules. Indeed, zinc is found in a large number of enzymes and other proteins, where it plays an important structural role [22].

The phytate contents before and after treatments are shown in Table 2. Phytate content varied from 556.52 to 606.07 mg/100 g DW of raw sorghum. These values are close to those reviewed by Greiner and Konietzny [24] and Kayodé [4] whom found that sorghum phytate ranged from 590 to 1180 and from 400 to 3500 mg/100 g dwt. Depending on the amount of plant derived foods in the diet and the grade of food processing, the daily intake of phytate can be as high as 4500 mg. On average, daily intake of phytate was estimated to be 2000–2600 mg for vegetarian diets as well as diets of inhabitants of rural areas in developing countries and 150–1400 mg for mixed diets [25].

**Table 2:** Changes in phytate content, total phosphorus (TP) and phytate phosphorus (PP) during soaking and germination of whole grains*.

Treatments	Phytate contentmg/100 g dw	% Phytate loss	Total phosphorusmg/100 g dw	Phytate phosphorusmg/100 g dw	PercentagePP/TP
**Raw**					
Dorado	591.00±14.45^ab^	-	376.09±12.33^a^	169.69±4.14^ab^	45.12
Shandaweel-6	606.07±34.64^a^	-	334.46±1.89^b^	174.01±9.94^a^	52.03
Giza-15	556.52±15.83^b^	-	381.37±23.02^a^	159.79±4.54^b^	41.90
**Soaking**					
Dorado	425.86±4.30^c^	27.94	358.65±12.84^a^	122.43±1.26^c^	34.14
Shandaweel-6	409.71±15.92^c^	32.40	275.75±5.39^d^	117.63±4.57^c^	42.66
Giza-15	425.26±13.83^c^	23.59	300.73±20.42^c^	122.10±3.97^c^	40.60
**Germination**					
Dorado	421.21±13.85^c^	28.73	235.50±18.62^e^	120.94±3.98^c^	51.35
Shandaweel-6	392.31±33.83^c^	35.27	203.14±4.43^f^	112.64±9.71^c^	55.45
Giza-15	417.85±13.56^c^	24.92	275.55±7.80^d^	119.97±3.89^c^	43.54
**L.S.D**	34.5136		23.7418	9.9096	

*Values are mean of three replicates ±SD, number in the same column followed by the same letter are not significantly different at *p*<0.05.

Weaning foods in developing countries are usually based on cereals, which contain phytate, a known inhibitor of iron and zinc absorption [26], [27], [28], [29], [30]. These phytate-containing foods may therefore be a strong contributing factor to poor iron and zinc status, which is commonly seen after 6 months of age, primarily in low-income countries but also in high-income countries [31], [32]. In a study from Malawi, a high intake of phytate was correlated with poor iron and zinc status in preschool children [33].

After soaking and germination there was a 23.59–32.4% and 24.92–35.27% decrease in phyate content, respectively. These findings are in range of the findings in previous studies found that soaking, germination, mashing, boiling and fermentation strongly reduced the phytate content and is more effective if whole grains are used [34], [35]. The magnitude of reduction induced by soaking in this study can be explained by the leaching in soaking medium or by partial hydrolysis by endogenous phytase. The reduction in phytate caused by soaking may be due to water solubilization of some phytic acid salts. Also, phytate content in the sorghum flour was significantly (*P*<0.05) reduced in all processed samples, eg soaking, boiling and fermentation [36]. In addition, germination activates endogenous grain phytase which can degrade phytate [37], [38]. During germination, phytins are broken down by endogenous phytase enzymes, releasing their P, myo-inositol (hereafter referred to as ‘inositol’) and mineral contents for use by the growing seedling [39].

As shown in Table 2, revealed that the values of total phosphorus of raw sorghum ranged from 334.46 to 381.37 mg/100 g DW. After soaking and germination the total phosphorus content was decreased from 275.75 to 358.65 and 203.14 to 275.55 mg/100 g dwt, respectively. Phytate phosporus ranged from 159.79 to 174.01 mg/100 g DW. These findings are in range of the findings by Radhakrishnan and Sivaprasad [40] and Godoy *et al*. [41].

### Effect of soaking and germination of whole grains on phytate (iron and zinc) molar ratios and phytases (acid and alkaline) activities

**Figure 1:**
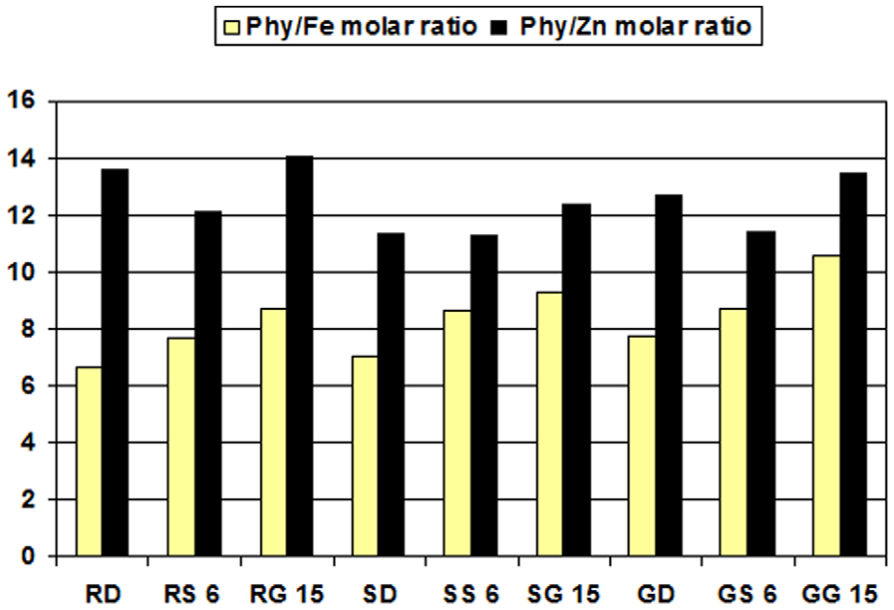
Effect of soaking and germination of whole seeds on phytate iron and zinc molar ratios. RD: Raw Dorado; RS 6: Raw Shandaweel-6; RG 16: Raw Giza-15; SD: Soaking Dorado; SS 6: Soaking Shandaweel-6; SG 15: Soaking Giza-15; GD: Germination Dorado; RS 6: Germination Shandaweel-6; GG 15: Germination Giza-15.

The effect of soaking and germination of raw sorghum on phyt/Fe and phyt/Zn molar ratio**s** were determined (Figure 1). Phyt/Fe and phyt/Zn molar ratio**s** were associated with iron and zinc absorbtion capacity. It could be noticed that the phyt/Fe molar ratio**s** ranged from 6.66 to 8.68 for raw sorghum. While the phyt/Zn ratio ranged from 12.16 to 14.08 in raw sorghum. Our conclusion proved that soaking and germination increased, the phyt/Fe molar ratio increased (7.06–9.23 and 7.74–10.57) while the phyt/Zn molar ratio decreased (11.29–12.38 and 11.43–13.44), respectively. In fact there was an increase in Phy/Fe molar ratio after soaking, because of the decrease in the iron content. After soaking the Phy/Zn molar ratios decreased slightly in almost all sorghum varieties [22]. These data confirm the report by Kayodé [4] who showed a phytate/Fe ratio lower than 14, which is the critical value above which Fe availability is strongly impaired. Our results reinforce previous results that showed that the bioavailability of zinc in cereals and legumes would be lower than that in vegetables and in some roots and tubers whose Phy/Zn molar ratios are generally less than 20 [42], [43]. Kayode *et al*. [44] calculated the phytate/Fe and phytate/Zn molar ratios as an index for the potential mineral bioavailability. Also, sorghum phytate was hydrolyzed during germination, so that iron solubility under simulated physiological conditions was greatly increased. It is somewhat difficult to predict the overall impact of soaking or germination on iron solubility. Soaking or germination might be effective in reducing the phytate content of white sorghum, especially if whole grains are used [45].

The activities of phytases (acid and alkaline) before and after treatments are shown in Table 3. The data showed significant differences between activity of acid and alkaline phytase and non significant increase in acid and alkaline phytase activities after soaking and germination. Phytase enzymes will be activated during drying in equal form in all varieties. Therefore the main distinct point is the change of phytase activity as will as specific activity during different treatment which showed no significant differences between the varieties used. These finding are in agreement with the findings of Marero *et al*. [46] who reported that phytate has been degraded in cereal foods by adding phytases or by activating endogenous phytase by a combination of soaking, germination and fermentation which is of a similar order of magnitude as observed by us. Also, humans have negligible intestinal phytase activity [47], even if they usually consume high phytate diets [48]. Cereals, however, contain an endogenous phytase. Because the endogenous cereal phytase has a pH optimum of 5.15, it is probably inactivated in the low pH of the stomach. Thus, there has been some interest in reducing the phytate content of cereals by soaking or germination (which activate endogenous phytase), or by adding a commercial phytase enzyme. Soaking under optimal conditions activates naturally occurring phytases in cereals and results in varying degrees of phytate hydrolysis depending on the kind of cereals [49].

**Table 3:** Effect of soaking and germination of whole grains on acid and alkaline phytase activity and specific activity*.

Treatments	Acid Phytase activity(unite/g dwt)	Alkaline Phytase activity(unite/g dwt)	L.S.D	Acid Phytase specific activity unit/mg protein	Alkaline Phytase specific activity unit/mg protein	L.S.D
**Raw**						
Dorado	1.005±0.045^a^	0.777±0.071^b^	0.1353	0.141±0.006^a^	0.110±0.01^b^	0.01914
Shandawee1-6	1.016±0.005^a^	0.781±0.006^b^	0.0530	0.116±0.001^a^	0.090±0.001^b^	0.00183
Giza-15	1.011±0.011^a^	0.797±0.005^b^	0.0733	0.125±0.001^a^	0.10±0.001^b^	0.0028
**Soaking**						
Dorado	1.020±0.03^a^	0.80±0.019^b^	0.0120	0.124±0.002^a^	0.096±0.002^b^	0.0044
Shandaweel-6	1.023±0.007^a^	0.788±0.071^b^	0.1136	0.113±0.001^a^	0.088±0.007^b^	0.01097
Giza-15	1.021±0.033^a^	0.798±0.003^b^	0.0141	0.126±0.004^a^	0.098±0.004^b^	0.0088
**Germination**						
Dorado	1.040±0.05^a^	0.825±0.005^b^	0.0187	0.080±0.003^a^	0.063±0.001^b^	0.0054
Shandaweel-6	1.020±0.006^a^	0.784±0.007^b^	0.0526	0.061±0.001^a^	0.050±0.001^b^	0.0024
Giza-15	1.030±0.040^a^	0.798±0.006^b^	0.0646	0.072±0.001^a^	0.055±0.001^b^	0.0022

*Values are mean of three replicates ±SD, number in the same column or raw followed by the same letter are not significantly different at *P*<0.05.

Most plant grains and seeds exhibit phytate-degrading activity over a wide pH range (pH = 3–10) [24] with maximal activity at pH values from pH = 5–5.5 [50]. Compared to legumes, cereals exhibit, in general, a significantly higher phytate-degrading activity in the pH range from pH = 5–5.5 [10], [11], whereas phytate-degrading activity at pH = 8.0 was slightly lower in cereals compared to legumes [24]. Performing activity assays by incubation of flours of grains and seeds at pH = 5.5 and defining 1 phytase unit (U) as equivalent to the enzymatic activity liberating 1 µmol of phosphate per minute, phytate-degrading activity in cereal seeds ranges from 0.10 to 7.0 U/g in [11] were shown. To understand phytate hydrolysis it is important to recognize and account not only for phytase activity, but also for activities of further phosphatases present in the plant material. Per definition all enzymes capable of dephosphorylating phytate are classified as phytases. However, myo-inositol pentakis-, tetrakis-, tris-, bis-, and monophosphates, the products of phytase action on phytate, might be further dephosphorylated during food processing by phytases as well as phosphatases which do not accept phytate as a substrate. Regarding the specific activity (unite/mg protein) data showed significant differences between activity of acid and alkaline phytase and non significant decrease in acid and alkaline phytase activities after soaking and germination.

### Effect of soaking and germination of whole seeds on *in vitro* iron and zinc bioavailability

*In vitro* iron and zinc bioavailability before and after soaking and germination are shown in Table 4. It could be noticed that the *In vitro* iron and zinc availability ranged between 8.02–13.60 and 7.35–9.73% for the raw sorghum, grains. The *In vitro* iron and zinc bioavailability after soaking and germination increased (14.62–20.75 and 9.07–10.72 for soaking treatment and 16.67–20.63 and 12.06–18.30 for germination treatment). The bioavailability of iron and zinc were significantly improved as a result of soaking and germination treatments especially for Giza-15 which was the highest varieties in bioavailability of iron during soaking and germination treatment and shandweel-6 which was the highest varieties in bioavailability of zinc during germination treatment. These finding are in agreement with the findings of Henriksen *et al*. [51] who reported that Food processing such as heat treatment, baking, fermentation, soaking, and milling may enhance or reduce iron availability. Also, phytase enzymes break down inositol hexa and penta phosphates, which inhibit iron absorption to smaller inositol phosphates and inorganic phosphate, which do not affect iron absorption. Soaking of wheat bran increased the soluble iron content from less than 5 percent to over 50 percent by destroying practically all their phytate thereby enhancing *in vitro* iron availability [49], [52]. Two common inhibitors of Fe absorption are tannins and phytate. These components form complexes with Fe within the intestinal lumen, reducing Fe bioavailability [5]. Some antinutritional factors chelate dietary mineral in the gastrointestinal tract reducing their bioaccessibility and bioavailability [53]. Processing techniques such as soaking, cooking, germination and fermentation have been found to reduce significantly the levels of phytates and tannins by exogenous and endogenous enzymes formed during processing [54]. Iron bioavailability is low due to high levels of dietary phytates and fibers in vegetarian diets [55]. Vegetarian meals have a poor bioavailability of zinc, and these diets may or may not have low zinc content [56]. At low zinc intakes and with an absence of inhibitors, zinc absorption can be greater than 50% [57]. Further, in Indian cooking processes, the main inhibitory factor of zinc bioavailability, phytate, gets partially degraded and may not remain as a strong inhibitor [58].

**Table 4:** Effect of soaking and germination of whole seeds on *in vitro* iron and zinc bioavailability*.

Treatments	*In vitro* iron availability %	*In vitro* zinc availability %
**Raw**		
Dorado	9.07±0.92^cd^	7.35±1.37^c^
Shandaweel-6	8.02±1.12^d^	8.87±0.09^bc^
Giza-15	13.16±0.73^bc^	9.73±2.87^bc^
**Soaking**		
Dorado	15.50±5.70^b^	10.23±4.19^bc^
Shandaweel-6	14.62±0.94^b^	9.07±0.52^bc^
Giza-15	20.75±1.20^a^	10.72±1.11^bc^
**Germination**		
Dorado	17.38±0.37^ab^	12.06±0.81^b^
Shandaweel-6	16.67±4.39^ab^	18.30±1.07^a^
Giza-15	20.63±2.84^a^	16.94±0.33^a^
**L.S.D**	4.6263	3.1928

*Values are mean of three replicates ±SD, number in the same column followed by the same letter are not significantly different at p<0.05.

## References

[1] Elkhalifa AO, Schiffler B, Bernhardt R (2005). Effect of fermentation on the functional properties of sorghum flour.. Food Chem.

[2] Hulse JH, Learning EM, Pearson OE (1980). Sorghum and the millets: Their composition and nutritive value.

[3] Serna-Saldivar S, Rooney LW, Dendy DAV (1995). Structure and chemistry of sorghum and millets.. Structure and chemistry of sorghum and millets.

[4] Kayodé APP (2006). Diversity, users' perception and food processing of sorghum: Implications for dietary iron and zinc supply..

[5] Elkhalil EAI, El-Tinay AH, Mohamed BE, Elsheikh EAE (2001). Effect of malt pretreatment on phytic acid and *in vitro* protein digestibility of sorghum flour.. Food Chem.

[6] Elsheikh EAE, Fadul IA, El Tinay AH (2000). Effect of cooking on anti-nutritional factors and *in vitro* protein digestibility (IVPD) of faba bean grown with different nutritional regimes.. Food Chem.

[7] Barrier GB, Casado P, Maupetit P, Jondreville C, Gatel F (1996). Wheat phosphorus availability: 2- *in vivo* study in broilers and pigs; relationship with indigenous phytase activity and phytic phosphorus content in wheat.. J Sci Food Agric.

[8] Muliman VH, Vadiraj S (1994). Changes in trypsin and chymotrypsin inhibitory activity on soaking of sorghum (*Sorghum bicolor* L. Moench).. Plant Foods Human Nutr.

[9] Greiner R, Konietzny U (1999). Improving enzymatic reduction of myo-inositol phosphates with inhibitory effects on mineral absorption in black beans (*Phaseolus vulgaris* var. Preto).. J Food Process Preserv.

[10] Egli I, Davidsson L, Juillerat MA, Barclay D, Hurrell RF (2002). The influence of soaking and germination on the phytase activity and phytic acid content of grains and seeds potentially useful for complementary feeding.. J Food Sci.

[11] Steiner T, Mosenthin R, Zimmermann B, Greiner R, Roth S (2007). Distribution of total phosphorus, phytate phosphorus and phytase activity in legume seeds, cereals and cereal by-products as influenced by harvest year and cultivar.. Anim Feed Sci Technol.

[12] Greiner R, Muzquiz M, Burbano C, Cuadrado C, Pedrosa MM (2001). Purification and characterization of a phytate degrading enzyme from germinated faba beans (*Vicia faba* var. Alameda).. J Agric Food Chem.

[13] Greiner R (2002). Purification and characterization of three phytate degrading enzymes from germinated lupin seeds (*Lupinus albus* var. Amiga).. J Agric Food Chem.

[14] AOAC (2000). Official Method of Analysis of the Association of official Analytical Chemists International, 17^th^ edition.

[15] Trough E, Mayer AH (1939). Improvement in the deiness calorimetric method for phosphorus and areseni.. Indian Eng Chem Annual.

[16] Mohamed A, Perera PJ, Hafez YS (1986). New chromophore for phytic acid determination.. Cereal Chem.

[17] Barrientos L, Scott JJ, Murthy PPN (1994). Specificity of hydrolysis of phytic acid by alkaline phytase from lily pollen.. Plant Physiol.

[18] Jog SP, Garchow BG, Mehta BD, Murthy PPN (2005). Alkaline phytase from lily pollen: Investigation of biochemical properties.. Archives Biochem Bioph.

[19] Lowry OH, Rosebrough NJ, Farr AL, Randall RJ (1951). Protein measurement with the folin phenol reagent.. J Biol Chem.

[20] Kiers JL, Nout MJR, Rombouts FM (2000). *In vitro* digestibility of processed and fermented soya bean, cowpea and maize.. J Sci Food Agric.

[21] Jambunathan R (1980). Improvement of the nutritional quality of sorghum and pearl millet.. Food Nutr Bulletin.

[22] Lestienne I, Icard-Verniére C, Mouquet C, Picq C, Tréche S (2005). Effects of soaking whole cereal and legume seeds on iron, zinc and phytate contents.. Food Chem.

[23] Saharan K, Khetarpaul N, Bishnoi S (2001). HCl-extractability of minerals from rice bean and faba bean: Influence of domestic processing methods.. Innovative Food Sci Emerging Tech.

[24] Greiner R, Konietzny U (2006). Phytase for Food Application.. Food Technol Biotechnol.

[25] Reddy NR, Reddy NR, Sathe SK (2002). Occurrence, Distribution, Content, and Dietary Intake of Phytate.. Food phytates.

[26] Lönnerdal B (2000). Dietary factors influencing zinc absorption.. J Nutr.

[27] Shehab GG, Kansowa OA, El-Beltagi HS (2010). Effects of various chemical agents for alleviation of drought stress in Rice plants (*Oryza sativa* L.).. Not Bot Hort Agrobot Cluj.

[28] Shallan MA, El-Beltagi HS, Mona A, Amera TM, Sohir NA (2010). Effect of amylose content and pre-germinated brown rice on serum blood glucose and lipids in experimental animal.. AJBAS.

[29] Shallan MA, El-Beltagi HS, Mona A, Amera TM (2010). Chemical Evaluation of Pre-germinated Brown Rice and Whole Grain Rice Bread.. EJEAFChe.

[30] El-Beltagi HS (2011). Effect of roasting treatments on protein fraction profiles, some enzyme activities of Egyptian peanuts.. Int J Food Sci Nutr.

[31] Gibson RS (1994). Zinc nutrition in developing countries.. Nutr Res Rev.

[32] Abdel-Rahim EA, El-Beltagi HS (2010). Constituents of apple, parsley and lentil edible plants and their therapy treatments for blood picture as well as liver and kidneys functions against lipidemic disease.. EJEAFChe.

[33] Lind T, Lönnerdal B, Persson L, Stenlund H, Tennefors C (2003). Effects of weaning cereals with different phytate contents on hemoglobin, iron stores, and serum zinc: a randomized intervention in infants from 6 to 12 mo of age1–3.. J Clin Nutr.

[34] Allen LH, Ahluwalia N (1997). Improving Iron Status Through Diet The Application of Knowledge Concerning Dietary Iron Bioavailability in Human Populations..

[35] Mahgoub SEO, Elhag SA (1998). Effect of milling, soaking, malting, heat-treatment and fermentation on phytate level of four Sudanese sorghum cultivars.. Food Chem.

[36] Towo E, Matuschek E, Svanberg U (2006). Fermentation and enzyme treatment of tannin sorghum gruels: effects on phenolic compounds, phytate and in vitro accessible iron.. Food Chem.

[37] Svanberg U, Lorri W (1997). Fermentation and nutrient availability.. Food Control.

[38] Kayode' APP, Hounhouigana JD, Nout MJR (2007). Impact of brewing process operations on phytate, phenolic compounds and in vitro solubility of iron and zinc in opaque sorghum beer.. LWT.

[39] Raboy V, Morre D, Boss W, Loewus F (1990). The biochemistry and genetics of phytic acid synthesis.. Inositol metabolism in plants.

[40] Radhakrishnan MR, Sivaprasad J (1980). Tannin content of sorghum verities and their role in iron bioavailability.. J Agric Food Chem.

[41] Godoy S, Chicco C, Meschy F, Requena F (2005). Phytic phosphorus and phytase activity of animal feed ingredients.. Communications Rep.

[42] Ferguson EL, Gibson RS, Thompson LU, Ounpuu S, Berry M (1988). Phytate, Zinc, and Calcium contents of 30 east African foods and their calculated phytate: Zn, Ca:Phytate and [Ca][Phytate]/[Zn] molar ratios.. J Food Comp Anal.

[43] Adeyeye EI, Arogundade LA, Akintayo ET, Aisida OA, Alao PA (2000). Calcium, zinc and phytate interrelationships in some foods of major consumption in Nigeria.. Food Chem.

[44] Kayode PAP, Linnemann AR, Nout MJR, Hounhouigan DJ, Stomph TJ (2006). Diversity and food quality properties of farmers' varieties of sorghum from Bénin.. J Sci Food Agric.

[45] Svanberg U, Sandberg A, Alnwick D, Moses S, Schmidt OG (1987). “Improved iron availability in weaning foods through the use of germination and fermentation.”. Improving Young Child Feeding in Eastern and Southern Africa: Proceedings of a Workshop in Nairobi, Kenya, October 1987.

[46] Marero LM, Payumo EM, Aguinaldo AR, Matsumoto I, Homma S (1991). The antinutritional factors in weaning foods prepared from germinated legumes and cereals.. Lebensmittelwissenschaft und Tech.

[47] Iqbal TH, Lewis KO, Cooper BT (1994). Phytase activity in the human and rat small intestine.. Gut.

[48] Brune M, Rossander L, Hallberg L (1989). Iron absorption: No intestinal adaptation to a highphytate diet.. Am J Clin Nutr.

[49] Sandberg AS, Svanberg U (1991). Phytate hydrolysis by phytase in cereals: Effects on *in vitro* estimation of iron availability.. J Food Sci.

[50] Konietzny U, Greiner R (2002). Molecular and catalytic properties of phytate-degrading enzymes (phytases).. Int J Food Sci Tech.

[51] Henriksen LK, Mahalko JR, Johnson LAK (1985). Canned foods: Appropriate in trace element studies?.. J Am Dietet Assoc.

[52] Hallberg L, Brune M, Rossander L (1989). Iron absorption in man: Ascorbic acid and dose-dependent inhibition by phytate.. Am J Clin Nutr.

[53] Frolich W (1995). Bioavailability of micronutrients in a fiber-rich diet, especially related to minerals.. Eu J Clin Nutr.

[54] Mosha MC, Svanberg U (1990). The acceptance and food intake of bulk reduced weaning. The Liganga village study.. Food Nutr Bull.

[55] Troost FJ, Brummer RJ, Dainty JR, Hoogewerff JA, Bull VJ (2003). Iron supplements inhibit zinc but not copper absorption in vivo in ileostomy subjects.. Am J Clin Nutr.

[56] Agte VV, Tarwadi KV, Chiplonkar SA, Roussel AM, Anderson RA, Favier AE (2000). The influence of various food ingredients and their combinations on in vitro availability of iron and zinc in cereal-based vegetarian meals.. Trace elements in man and animals, volume 10.

[57] Sandstrom B (1992). Dose dependence of zinc and manganese absorption in man.. Proc Nutr Soc.

[58] Agte VV, Tarwadi KV, Chiplonkar SA (1999). Phytate degradation during raditional cooking: significance of the phytic acid profile in cerealbased vegetarian meals.. J Food Comp Anal.

