# Expression of concern: Bioavailability of iron, zinc, phytate and phytase activity during soaking and germination of white sorghum varieties

**DOI:** 10.1371/journal.pone.0351800

**Published:** 2026-06-17

**Authors:** The PLOS One Editors

The *PLOS One* Editors issue this Expression of Concern for this article [1] because of concerns identified about the peer review process. Readers are advised to interpret the article [1] with caution.
